# A noninferiority within-person study comparing the accuracy of transperineal to transrectal MRI–US fusion biopsy for prostate-cancer detection

**DOI:** 10.1038/s41391-020-0205-7

**Published:** 2020-01-17

**Authors:** Yaara Ber, Niv Segal, Shlomit Tamir, Ofer Benjaminov, Maxim Yakimov, Sivan Sela, Daniel Halstauch, Jack Baniel, Daniel Kedar, David Margel

**Affiliations:** 1 Division of Urology, Rabin Medical Center, Petah-Tikva, Israel; 2 Department of Surgery, Sackler Faculty of Medicine, Tel Aviv University, Tel Aviv-Yafo, Israel; 3 Department of Imaging, Rabin Medical Center, Petah-Tikva, Israel; 4 Division of Imaging, Shaare Zedek Medical Center, Jerusalem, Israel; 5 Department of Pathology, Rabin Medical Center, Petah-Tikva, Israel

**Keywords:** Prostate cancer, Prostate cancer

## Abstract

**Background:**

Magnetic resonance imaging (MRI) and ultrasound (US) fusion prostate-biopsies can be performed in a transrectal (TR-fusion) or transperineal (TP-fusion) approach. Prospective comparative evidence is limited. In this study we compared the detection rate of clinically-significant prostate-cancer (csPCa) within an index lesion between TR and TP-fusion.

**Patients and methods:**

This was a prospective, noninferiority, and within-person trial. Men scheduled for MRI–US-fusion with a discrete MRI PI-RRAD ≥ 3 lesion were included. A dominant index lesion was determined for each subject and sampled by TR and TP-fusion during the same session. The order of biopsies was randomized and equipment was reset to avoid chronological and incorporation bias. For each subject, the index lesion was sampled 4–6 times in each approach. All biopsies were performed using Navigo fusion software (UC-Care, Yokneam, Israel). csPCa was defined as: Grade Group ≥ 2 or cancer-core length ≥ 6 mm. We used a noninferiority margin of 10% and a one-sided alpha level of 5%.

**Results:**

Seventy-seven patients completed the protocol. Median age was 68.2 years (IQR:64.2–72.2), median PSA was 8.9 ng/ml (IQR:6.18–12.2). Ten patients (13%) were biopsy naive, others (87%) had a previous biopsy. csPCa was detected in 32 patients (42%). All of these cases were detected by TP-fusion, while only 20 (26%) by TR-fusion. Absolute difference for csPCa diagnosis was 15.6 (CI 90% 27.9–3.2%) in favor of TP-fusion (*p* = 0.029). TP-fusion was noninferior to TR-fusion. The lower boundary of the 90% confidence-interval between TP-fusion and TR-fusion was greater than zero, therefore TP-fusion was also found to be superior. Exploratory subgroup analyses showed TP-fusion was consistently associated with higher detection rates of csPCa compared with TR-fusion in patient and index-lesion derived subgroups (size, location, PI-RADS, PSA, and biopsy history).

**Conclusions:**

In this study, TP-fusion biopsies were found to be noninferior and superior to TR-fusion biopsies in detecting csPCa within MRI-visible index lesion. Centers experienced in both TP and TR-fusion should consider these results when choosing biopsy method.

## Introduction

Prostate-cancer diagnosis is undergoing a paradigm shift. Data from the PROMIS study demonstrated that multiparametric magnetic resonance imaging (mpMRI), before prostate biopsy, could identify men who might safely avoid biopsy and improve detection of clinically-significant cancer [1]. The PRECISION trial demonstrated that MRI–ultrasound (US)-fusion biopsies are superior to systematic transrectal US-guided (TRUS) biopsies for prostate-cancer detection among biopsy-naive patients [2].

MRI–US fusion biopsies, like systematic biopsies, can be performed transrectally or transperineally. The transrectal (TR) approach is typically performed under local anesthesia, and is associated with about 3% of septic complications [3]. The transperineal (TP) approach is usually performed under general anesthesia, thus is more time and resource consuming. However, septic complications are extremely rare [4].

In systematic biopsies, there are conflicting results as to whether the biopsy approach affect prostate-cancer detection rate. While several studies found that TP is preferable [5, 6], others suggest that there are no significant differences [7–9].

As MRI–US fusion biopsies are becoming standard of care, the TP versus TR dilemma becomes topical. To date, no study has prospectively compared the two methods in the fusion era. Here we present the results of noninferiority, within subject, study comparing TR-fusion and TP-fusion for diagnosis of clinically-significant prostate-cancer (csPCa).

## Patients and methods

### Trial design

We conducted a prospective, noninferiority, and within-subject trial comparing TR and TP-fusion of a MRI-visible index lesion. Each index lesion was sampled by TR and TP-fusion; the order of approach was randomized. This study was approved by the local ethics committee.

### Participants

We included patients referred to a MRI–US fusion prostate biopsy. All patients had mpMRI of the prostate with a discrete index lesion with PI-RADS V2 score ≥3. We took a pragmatic approach and included men undergoing either primary or repeated biopsy. We also included men who were previously diagnosed with low-risk prostate-cancer (Gleason Group 1) and are under active-surveillance protocol. Consecutive eligible patients providing written consent were enrolled.

### mpMRI and index lesion

We included patients who had a recent prostate-mpMRI (within 6 months) with at least three sequences-triplanar T2-weighted, dynamic contrast-enhanced, and diffusion-weighted imaging. MRI were scored according to PI-RADS v2 [10]. Two experienced uro-radiologists reviewed all MRIs and indicated the index lesion. The index lesion was defined as the MRI lesion with the highest PI-RADS or the largest lesion in cases of more than one lesion with the same PI-RADS.

### Intervention

All participants underwent MRI–US fusion prostate biopsy. We targeted the index lesion using TR-fusion and TP-fusion, during the same session. We randomized participants after anesthesia to decide which approach will be taken first: TP-fusion followed by a TR-fusion, or vice-versa. Patients were unaware of biopsies order. Randomization was performed to avoid chronological bias and to ensure that the biopsies order will not affect detection rate. All biopsies were done under general anesthesia using NaviGo™ fusion system (UC-Care, Yokneam, Israel). This device is approved for TR and TP-fusion biopsies. TR-fusion was performed in the left-decubitus position, to mimic outpatient clinic settings using a sidefire TRUS probe (BK flex 500, Germany). TP-fusion was performed in a high-lithotomy position, using a standard brachytherapy template.

MRI segmentation of the prostate and index-lesion contour was preformed prior to surgery. The same MRI segmentation was used for TP-fusion and TR-fusion. All biopsies were performed by the same operator (DM). The index lesion was sampled 4–6 times in TP-fusion and TR-fusion. To minimize incorporation bias, the alignment software was reset between approaches so that the overlay between the MRI and US to create the 3D-prostate model was done independently for each approach.

In addition, all patients underwent systematic biopsies in the contralateral lobe, opposite the index lesion (six cores) and sampling of any existing additional nonindex MRI lesions (4–6 cores) during the TP-fusion biopsy session. Biopsy samples were evaluated by a specialist uro-pathologist. The pathologist was blinded to the type of biopsy approach.

### Primary outcome

To compare the detection rate of csPCa within the index lesion between TP-fusion and TR-fusion biopsies. We defined csPCa as Grade Group (GG) ≥ 2 or cancer-core-length of 6 mm or more in any location [11–14].

### Secondary outcome

1. To compare the detection rate of prostate-cancer within the index lesion between TP and TR-fusion.
2. To compare the detection rate of GG ≥ 2 within the index lesion between TP and TR-fusion.
3. To compare the detection rate of csPCa between TP and TR-fusion biopsies, stratified by patient derived parameters (prostate size, previous prostate-biopsy history, PSA) and index-lesion parameters (location, PI-RADS, and size).

### Randomization

Participants were randomized in a 1:1 ratio. Randomization was done by minimization using the computerized “MINIM” software [15]. MRI and index lesion were segmented prior to randomization. Randomization was performed by an independent member of the study, after anesthesia. Patients and data analysist were blinded.

### Statistical analysis

#### Sample size

Based on data from our series, cancer detection rate of MRI–US fusion was 49% [16]. Using a noninferiority margin of 10% (this was decided based on clinically meaningful difference) and a one-sided alpha level of 5%, we calculated that randomization of 76 men (76 TR-fusion and 76 TP-fusion) would provide the trial with 80% power to show the noninferiority of the TP approach.

#### Outcome analyses

For the primary outcome, we calculated the csPCa detection rate in both approaches. If the lower boundary of the two-sided 90% confidence-interval (CI) for the absolute difference in csPCa rates between TP-fusion relative to TR-fusion was greater than −10 percentage points, then TP-fusion would be deemed to be noninferior. Furthermore, if the lower boundary was greater than zero, superiority would be claimed.

As this was a noninferiority trial, the per-protocol analysis was the primary analysis. We also included an intention-to-treat analysis using all randomized participants. The per-protocol analysis included only men who underwent TP-fusion and TR-fusion during the same session.

The study design included sampling the same lesion within the same patient by both approaches. Thus, we also performed a paired analysis within subject to compare the two approaches using Mcnemar test.

Exploratory subgroup analysis comparing the effect of baseline parameters was done with odds ratio and 95% CI.

All statistical analysis was performed using IBM SPSS statistics ver21.00. A *p* value < 0.05 was considered statistically significant.

## Results

### Patients characteristics

**Fig. 1:**
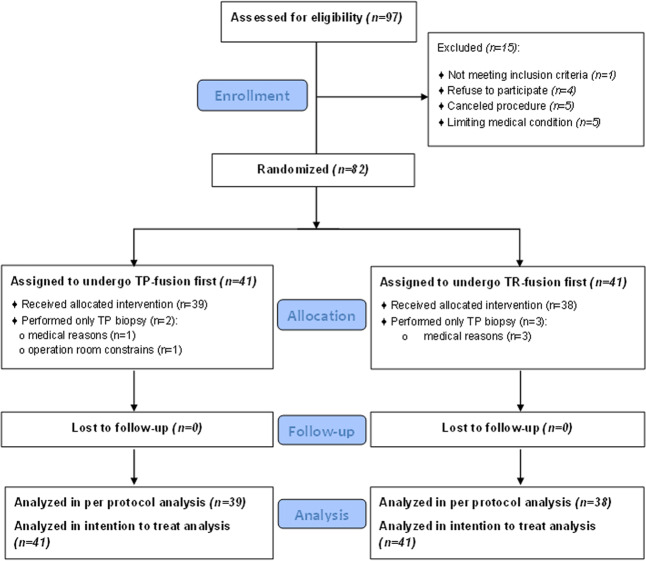
Enrollment, randomization, and analysis.

Between December 2017 and July 2018, we randomized 82 participants, scheduled for MRI–US fusion prostate biopsy in Rabin-Medical-Center, Israel. A total of 77 participants underwent the full protocol, including TP-fusion and TR-fusion of the index lesion, during the same session (Fig. 1).

Participant’s median age was 68.2 years (IQR 64.2–72.2), and median PSA was 8.9 ng/ml (IQR 6.2–12.2). Sixty-seven participants (87%) had a previous biopsy and 23 (30%) were previously diagnosed with low-risk prostate-cancer. All participants had a suspected lesion in mpMRI: 35 (45%) with PI-RADS-3 lesion, 32 (42%) with PI-RADS-4, and 10 (13%) with PI-RADS-5 (Tables 1 and S1).

**Table 1:** Characteristics of participants at baseline.

	All (*N* = 77)
Age (years), median (IQR)	68.2 (64.2–72.2)
PSA ng/ml, median (IQR)	8.9 (6.2–12.2)
Prostate volume cm^3^, median (IQR)	53 (40–82)
PSA density, median (IQR)	0.14 (0.09–0.27)
Biopsy naive, no. (%)	10 (13%)
Previous negative biopsies, no. (%):	
1	22 (29%)
2	15 (19%)
3 and up	7 (9%)
Active surveillance, no. (%)^a^	23 (30%)
Family history of PCa, no. (%)	13 (17%)
PI-RADS no. (%)	
3	35 (45%)
4	32 (42%)
5	10 (13%)
Index-lesion location, axial view, no. (%)	
Apex	42 (54.6%)
Base	9 (11.7%)
Midgland	26 (33.8%)
Index-lesion location, coronal view, no. (%)	
Peripheral	46 (59.7%)
Transition zone	18 (23.4%)
Anterior	13 (16.9%)
Ellipsoid index-lesion volume, median (IQR)^b^	0.39 (0.19–1.07)

*PCa* prostate cancer, *PI*-*RADS* prostate imaging reporting and data system, *PSA* protein specific antigen

^a^All participants under active-surveillance protocol has previous biopsy with GG1

^b^Ellipsoid index-lesion volume = 4Π/3 × height × length × width

### Clinically significant prostate-cancer detection rate

**Fig. 2:**
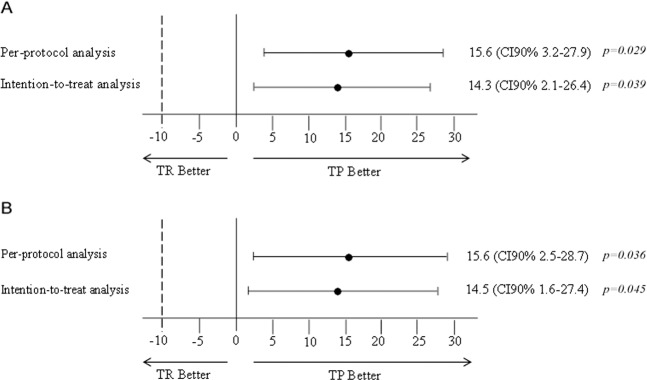
Per-protocol and intention-to-treat analyses for the detection of clinically significant prostate cancer and all prostate cancer. Shown are the absolute differences between TP-fusion and TR-fusion clinically significant (**a**) and all prostate cancer (**b**) detection rates. The per-protocol analysis included all patients who completed index-lesion biopsy by TR-fusion and TP-fusion, as specified in the protocol. Intention-to-treat analysis included all the participants who underwent randomization. If the lower boundary of the two-sided 90% confidence-interval for the difference was greater than −10 percentage points (dashed line), then TP-fusion would be deemed to be noninferior. If the lower boundary was greater than zero (solid line), superiority would be claimed. TP transperineal, TR transrectal.

csPCa was detected in 32 participants (41.6%). All of these cases were detected by TP-fusion, while only 20 cases (26%) were detected by TR-fusion (Table 2). The absolute difference for csPCa detection rate in the per-protocol analysis was 15.6 (CI 90% 3.2–27.9%) in favor of TP-fusion (*p* = 0.029) (Fig. 2a). The lower boundary of the 90% CI for the difference was greater than −10 percentage points, demonstrating that TP-fusion is noninferior to TR-fusion in detection of csPCa. Furthermore, since the lower boundary of the 90% CI was greater than zero, superiority of TP-fusion was demonstrated (Fig. 2a).

**Table 2:** Prostate-cancer detection in index lesion.

	TP-fusion (*N* = 77)	TR-fusion (*N* = 77)
csPCa detection^a^, no. (%)	32 (41.6%)	20 (26%)
Grade group, no. (%)		
GG1	22 (28.6%)	16 (20.8%)
GG2	12 (15.6%)	11 (14.3%)
GG3	4 (5.2%)	2 (2.6%)
GG4	3 (3.9%)	2 (2.6%)
GG5	3 (3.9%)	1 (1.3%)
No cancer	33 (42.9%)	45 (58.4%)
Total cancer detection, no. (%)	44 (57.1%)	32 (41.6%)
Total cancer cores length^b^ (mm), median (IQR)	10 (3–19)	7 (2–13)
Number of cores taken, median (IQR)	5 (4–6)	5 (4–6)
Percentage of positive cores^c^, % (IQR)	66% (24%–85%)	59% (23%–76%)
GG2 ≥ 2, no. (%)	22 (28.6%)	16 (20.1%)
GG3 ≥ 3, no. (%)	10 (13%)	5 (6.5%)

*csPCa* clinically significant prostate cancer, *GG* grade group, *TP* transperineal, *TR* transrectal

^a^csPCa was defined as GG ≥ 2 or cancer core length of 6 mm or more

^b^Total cancer length was calculated as the sum as all cancerous segments from highest GG

^c^Percent of positive cores refers only to cores taken from index lesion

TP-fusion remained superior to TR-fusion also in the intention-to-treat analysis (absolute difference 14.3, CI 90% 2.1–26.4%, *p* = 0.039) (Fig. 2a). The order of biopsy approach did not affect csPCa detection rate (Table S2).

The setting of our study allows comparing the two approaches, within each subject, using a matched-paired analysis. The TP-fusion approach did not miss any csPCa within the index lesion, while TR-fusion missed 12 cases (*p* < 0.0001, Table 3).

**Table 3:** Paired analysis of cancer detection rate.

	TP-fusion of index lesion		
	Negative	Positive	Total
csPCa^a^ (*p* < 0.0001)			
TR-fusion of index lesion			
Negative	45	12	57
Positive	0	20	20
Total	45	32	77
All detected cancer (*p* = 0.004)			
TR-fusion of index lesion			
Negative	31	14	45
Positive	2	30	32
Total	33	44	77
GG ≥ 2 (*p* = 0.07)			
TR-fusion of index lesion			
Negative	54	7	61
Positive	1	15	16
Total	55	22	77
GG ≥ 3 (*p* = 0.06)			
TR-fusion of index lesion			
Negative	67	5	72
Positive	0	5	5
Total	67	10	77

*csPCa* clinically significant prostate cancer, *GG* grade group, *TP* transperineal, *TR* transrectal

^a^csPCa was defined as GG ≥ 2 or cancer core length of 6 mm or more

### Prostate-cancer detection rate

Prostate cancer (significant and nonsignificant) was detected in 47 patients (61%). We detected 44 (57.1%) prostate-cancer cases by TP-fusion, and 32 (41.6%) by TR-fusion (Table 2). Noninferiority and superiority of TP-fusion was shown in per-protocol (absolute difference 15.6 (CI 90% 28.7–2.5%), *p* = 0.036) and in the intention-to-treat analysis (absolute difference 14.5 (CI 90% 27.4–1.6%), *p* = 0.045) (Fig. 2b). The order of biopsy approach did not affect PCa detection rate (Table S2).

This result is further supported by paired analysis. TP-fusion missed two cancer cases in the index lesion that were detected by TR-fusion. However, 14 cases that were detected by TP-fusion were missed by TR-fusion (*p* = 0.004, Table 3). Both cancer core length (*p* = 0.002) and percent of cores involved (*p* = 0.013) were higher in the TP-fusion (Table 2).

One case of prostate cancer (GG1, 1.3%) was missed by index-lesion sampling and detected by sampling of nonindex lesion. Results of nonindex-lesions and systematic biopsies are detailed in Table S3.

### Detection of grade group 2 and above

We detected GG ≥ 2 prostate cancer in 24 participants (31.1%). TP-fusion detected 22 cases (28.6%), within the index lesion. TR-fusion detected only 16 cases (20.1%) (Table 2). Only two GG2 cancers were missed by sampling the index lesion using the TP-fusion approach (one was detected only by systematic biopsy, and the other had a high-volume GG1). On the other hand, eight cases were missed sampling the index lesion using the TR-fusion approach.

Paired analysis showed a similar trend, though not statistically significant. TP-fusion missed one GG2 (this patient was diagnosed with a high-volume GG1 by TP-fusion) and no GG3 or above prostate cancers, while TR-fusion missed seven GG2 cancers and five GG3 or above cancers (Table 3).

### Subgroup analyses

In an exploratory subgroup analyses we found that TP-fusion was consistently associated with higher detection rates of csPCa compared with TR-fusion. All baseline characteristics including patient derived (prostate size, previous prostate-biopsy history, PSA), and index-lesion derived (location, size, and PI-RADS) showed preferability of the TP-fusion (Fig. S1).

### Adverse events

We followed all subjects for 8 weeks after the biopsy. Three (3.9%) adverse events were documented. One participant had bradycardia following biopsy, and was monitored in-hospital for 24 h. Other adverse events included one case of urinary retention, and one case of urinary tract infection. All events were resolved with no clinical consequences.

## Discussion

We performed a noninferiority, within-person study, comparing the detection rate of csPCa between TR and TP-fusion biopsies. Sampling the index lesion using the TP-fusion approach was found to be noninferior and the 90% CI indicated its superiority to TR-fusion. This was consistent in the per-protocol and in the intention-to-treat analyses. Furthermore, the paired analysis supported this conclusion. Exploratory subgroup analyses suggest that this conclusion was robust across participant and index-lesion derived parameters.

MRI–US fusion biopsies require a multidisciplinary cooperative effort including MRI technicians, interpreting radiologists, image segmentation, and the operating urologist. An error in each of these components may affect the accuracy of targeting.

Our study design mitigated many of the potential biases. The same radiologist interpretation and MRI segmentation was used in both techniques, thus MRI errors cannot account for the differences we found between TP and TR-fusion. The overlay between MRI and US to create 3D-prostate model could potentially include a deviation. However, in our study, we used the same MRI–US fusion system for both approaches, so any deviation error will equally affect both. We also checked for overlay asymmetry and did not find significant differences (data not shown).

In a previous study we demonstrated the effect of learning curve on detection rate; however, the performing surgeon was well experienced in both techniques [16].

Needle delivery may also have a significant impact on targeted biopsy. Our group showed that needle deflection occurs during TR biopsies [17]. As TP biopsies are performed via a stabilized template it is rational to assume less deflection, however, to the best of our knowledge, there are no in vivo study comparing deflection rate differences between the two approaches.

Our “real-world” population may introduce several biases. First we included many participants with a previous negative systematic biopsy, and only few who were biopsy naive. The anatomical locations of most missed PCa are in the apex, dorsolateral, and anterior prostate segments [18]. These are exactly the locations for which the TP route provides an easier access [19]. Second, including previously biopsied patients may select for patients with lower likelihood of clinically significant disease. Larger, higher grade lesions may be more likely to be detected with both TP and TR approaches. However, in our exploratory subgroup analyses TP was consistently more accurate than TR irrespective of index-lesion location or size, and irrespective of previous biopsy history.

We believe the best explanation for the cancer detection rate difference we observed is rooted in spatial alignment between tumor-3D configuration and needle trajectory. The largest diameter of most prostate tumors is along the longitudinal axis (apex to base) [20]. The TP needle is inserted along the same axis. Contrarily, in TR biopsies the needle penetrates through anterior–posterior axis and thus even targeted biopsies are centered at narrower axis. This is underscored in the differences in median tumor core length (10 mm in TP-fusion vs. 7 mm in TR-fusion). Sampling larger tumor volume may also improve detection of higher grade tumors, reducing sampling errors associated with tumor heterogeneity [21].

Be the reason as it may, others have also found that TP-fusion have a better detection rate than TR-fusion. Stabile et al. [22] in a multivariable-logistic-regression model predicting csPCa among 157 patients undergoing fusion biopsies found that TP-fusion was four times more likely to detect csPCa even controlling for PSA, prostate volume, and PI-RADS.

Pepe et al. [23] compared TR-fusion to TP-cognitive targeted biopsies. TP-cognitive approach identified more csPCa than TR-fusion. Tewes et al. [24] observed a higher detection rate for TP-fusion (75%) compared with TR-fusion (39%). None of the former studies performed a within patient analysis of the same index lesion.

The SmartTarget-biopsy trial [25] conducted a within-person prospective study comparing the accuracy of visual registration vs. fusion, both in TP position. Detection rate for TP-fusion (62%) were similar to ours. The FUTURE trial [26] compared between three MRI-targeted modalities: TP-fusion, TR-cognitive, and TR-in-bore MRI. Authors found no difference between the modalities in csPCa detection rate. However, they used a parallel group design and compared very different fusion systems. Thus, limiting the ability to determine differences between TP and TR-fusion. In our study we used the same fusion system and the same index lesion for TR and TP techniques, so technical limitations were equal between the two approaches, thus strengthening our results.

The TP-biopsy is performed via the perineal skin, thus avoiding needle passage through the rectal mucosa. A systematic review of TP-biopsies reported a negligible rate of infection-related hospitalizations with TP-biopsies [4]. On the other hand, infection rates following TR-biopsies are increasing [27, 28]. In our study, we cannot separate between TR and TP related adverse events. Overall we had one case of urinary retention, and urinary tract infection, none of which required hospital readmissions, similar to previously reported complications [4, 29].

Our study has several limitations. First, all biopsies were performed by a single surgeon (DM), using the same fusion system. However this within person, single operator design, may even strengthen our results. Since all other settings were equal, the differences between cancer detection rates can only be attributed to biopsy approach. Targeting the same index lesion may increase the detection rate of the second biopsy attempt. To avoid this bias we reset the fusion software between the two approaches, so that the 3D-model building was done independently for each approach. Moreover, we randomized the approach to be taken first.

Randomization also minimized the risk that hematoma or other deformities resulting from the first biopsy, or from the systematic biopsy taken during the TP-fusion, may reduce the possibility of cancer detection in the second approach. We took systematic biopsies only during the TP-fusion to reduce the risk of infection. This may have caused a bias in favor of TP when performed first, as the prostate might have altered by a systematic biopsy and targeted biopsy done using TP approach. However, we demonstrated that the order of biopsy had no effect on cancer detection in our trial (Table S2).

The generalizability of our results may be hampered by our single-fusion system and operator study design. However, our group and others did not observe a difference between different fusion systems [1, 16, 26]. In addition, our detection rates are comparable to other multicenter fusion studies [1, 2, 16, 26]. Our results apply to experienced centers where both TP and TR-fusion are performed routinely. We used a sidefire probe for TR-fusion that may have affected our results. Studies comparing prostate-cancer detection rate showed no difference between end and sidefire probes [30].

In conclusion, in our study TP-fusion was noninferior and superior to TR-fusion in identifying csPCa within the index lesion. This was robust in all subgroup analyses. However, this was a single-center, single-fusion system study, and requires confirmation in a multicenter setting.

## Supplementary information

Supplementary data

Supplementary Figure S1

## References

[1] Ahmed HU, El-Shater Bosaily A, Brown LC, Gabe R, Kaplan R, Parmar MK (2017). Diagnostic accuracy of multi-parametric MRI and TRUS biopsy in prostate cancer (PROMIS): a paired validating confirmatory study. Lancet..

[2] Kasivisvanathan V, Rannikko AS, Borghi M, Panebianco V, Mynderse LA, Vaarala MH (2018). MRI-targeted or standard biopsy for prostate-cancer diagnosis. N Engl J Med.

[3] Bruyère F, Malavaud S, Bertrand P, Decock A, Cariou G, Doublet JD (2015). Prosbiotate: a multicenter, prospective analysis of infectious complications after prostate biopsy. J Urol..

[4] Grummet JP, Weerakoon M, Huang S, Lawrentschuk N, Frydenberg M, Moon DA (2014). Sepsis and ‘superbugs’: should we favour the transperineal over the transrectal approach for prostate biopsy?. BJU Int.

[5] Emiliozzi P, Corsetti A, Tassi B, Federico G, Martini M, Pansadoro V (2003). Best approach for prostate cancer detection: a prospective study on transperineal versus transrectal six-core prostate biopsy. Urology..

[6] Mabjeesh NJ, Lidawi G, Chen J, German L, Matzkin H (2012). High detection rate of significant prostate tumors in anterior zones using transperineal ultrasound-guided template saturation biopsy. BJU Int.

[7] Takenaka A, Hara R, Ishimura T, Fujii T, Jo Y, Nagai A (2008). A prospective randomized comparison of diagnostic efficacy between transperineal and transrectal 12-core prostate biopsy. Prostate Cancer Prostatic Dis.

[8] Xiang J, Yan H, Li J, Wang X, Chen H, Zheng X (2019). Transperineal versus transrectal prostate biopsy in the diagnosis of prostate cancer: a systematic review and meta-analysis. World J Surg Oncol.

[9] Xue J, Qin Z, Cai H, Zhang C, Li X, Xu W (2017). Comparison between transrectal and transperineal prostate biopsy for detection of prostate cancer: a meta-analysis and trial sequential analysis. Oncotarget..

[10] Weinreb JC, Barentsz JO, Choyke PL, Cornud F, Haider MA, Macura KJ (2016). PI-RADS prostate imaging—reporting and data system: 2015, version 2. Eur Urol.

[11] Kepner G, Kepner J (2010). Transperineal biopsy: analysis of a uniform core sampling pattern that yields data on tumor volume limits in negative biopsies. Theor Biol Med Model.

[12] Wolters T, Roobol MJ, van Leeuwen P, van den Bergh RC, Hoedemaeker RF, van Leenders GJ (2011). A critical analysis of the tumor volume threshold for clinically insignificant prostate cancer using a data set of a random screening trial. J Urol.

[13] Stamey T, Freiha F, McNeal J, Redwine EA, Whittemore AS, Schmid HP (1993). Localized prostate cancer. Relationship of tumor volume to clinical significance for treatment of prostate cancer. Cancer.

[14] Ahmed HU, Hu Y, Carter T, Arumainayagam N, Lecornet E, Freeman A (2011). Characterizing clinically significant prostate cancer using template prostate mapping biopsy. J Urol.

[15] Evans S, Royston P, Day S. Minim: allocation by minimisation in clinical trials (ver 1.5). http://www-users.york.ac.uk/~mb55/guide/minim.htm. Accessed 27 Sep 2018.

[16] Halstuch D, Baniel J, Lifshitz D, Sela S, Ber Y, Margel D. Characterizing the learning curve of MRI–US fusion prostate biopsies. Prostate Cancer Prostatic Dis. 2019;22:546–51. 10.1038/s41391-019-0137-2 30842585

[17] Halstuch D, Baniel J, Lifshitz D, Sela S, Ber Y, Margel D (2018). Assessment of needle tip deflection during transrectal guided prostate biopsy: implications for targeted biopsies. J Endourol..

[18] Schouten MG, van der Leest M, Pokorny M, Hoogenboom M, Barentsz JO, Thompson LC (2017). Why and where do we miss significant prostate cancer with multi-parametric magnetic resonance imaging followed by magnetic resonance-guided and transrectal ultrasound-guided biopsy in biopsy-naïve men?. Eur Urol..

[19] Giannarini G, Crestani A, Rossanese M, Ficarra V (2017). Multiparametric magnetic resonance imaging targeted biopsy for early detection of prostate cancer: all that glitters is not gold!. Eur Urol..

[20] Mai Z, Zhou Z, Yan W, Xiao Y, Zhou Y, Liang Z (2018). The transverse and vertical distribution of prostate cancer in biopsy and radical prostatectomy specimens. BMC Cancer..

[21] Cyll K, Ersvær E, Vlatkovic L, Pradhan M, Kildal W, Avranden Kjær M (2017). Tumour heterogeneity poses a significant challenge to cancer biomarker research. Br J Cancer.

[22] Stabile A, Dell’Oglio P, Gandaglia G, Fossati N, Brembilla G, Cristel G (2018). Not all multiparametric magnetic resonance imaging–targeted biopsies are equal: the impact of the type of approach and operator expertise on the detection of clinically significant prostate cancer. Eur Urol Oncol.

[23] Pepe P, Garufi A, Priolo G, Pennisi M (2017). Transperineal versus transrectal MRI/TRUS fusion targeted biopsy: detection rate of clinically significant prostate cancer. Clin Genitourin Cancer.

[24] Tewes S, Peters I, Tiemeyer A, Peperhove M, Hartung D, Pertschy S (2017). Evaluation of MRI/ultrasound fusion-guided prostate biopsy using transrectal and transperineal approaches. Biomed Res Int.

[25] Hamid S, Donaldson IA, Hu Y, Rodell R, Villarini B, Bonmati E (2019). The smarttarget biopsy trial: a prospective, within-person randomised, blinded trial comparing the accuracy of visual-registration and magnetic resonance imaging/ultrasound image-fusion targeted biopsies for prostate cancer risk stratification. Eur Urol..

[26] Wegelin O, Exterkate L, van der Leest, Kummer JA, Vreuls W, de Bruin PC (2019). The FUTURE trial: a multicenter randomised controlled trial on target biopsy techniques based on magnetic resonance imaging in the diagnosis of prostate cancer in patients with prior negative biopsies. Eur Urol..

[27] Anastasiadis E, van der Meulen J, Emberton M (2015). Hospital admissions after transrectal ultrasound-guided biopsy of the prostate in men diagnosed with prostate cancer: a database analysis in England. Int J Urol.

[28] Carignan A, Roussy J-F, Lapointe V, Valiquette L, Sabbagh R, Pépin J (2012). Increasing risk of infectious complications after transrectal ultrasound-guided prostate biopsies: time to reassess antimicrobial prophylaxis?. Eur Urol.

[29] Stefanova V, Buckley R, Flax S, Spevack L, Hajek D, Tunis A (2019). Transperineal prostate biopsies using local anesthesia: experience with 1287 patients. Prostate cancer detection rate, complications and patient tolerability. J Urol.

[30] Rom M, Pycha A, Wiunig C, Reissigl A, Waldert M, Klatte T (2012). Prospective randomized multicenter study comparing prostate cancer detection rates of end-fire and side-fire transrectal ultrasound probe configuration. Urology..

