# Supplementary material for: A noninferiority within-person study comparing the accuracy of transperineal to transrectal MRI–US fusion biopsy for prostate-cancer detection
Source: Prostate Cancer Prostatic Dis. 2020 Jan 17;23(3):449–56. doi: 10.1038/s41391-020-0205-7 (PMC7423592; doi:10.1038/s41391-020-0205-7)
Supplement: Supplementary file 1 — Supplementary data [file 41391_2020_205_MOESM1_ESM.docx]

**A non-inferiority within-person study comparing the accuracy of transperineal to transrectal MRI-US fusion biopsy for prostate-cancer detection**

Yaara Ber^1^ PhD, Niv Segal^1,2^ MD, Shlomit Tamir^3^ MD, Ofer Benjaminov^4^ MD, Maxim Yakimov^5^ MD, Sivan Sela^1^ BSc, Daniel Halstauch^1^ MD, Jack Baniel^1,2^ MD, Daniel Kedar^1^ MD, David Margel^1,2^ MD, PhD

^1^ Division of Urology, Rabin Medical Center, Petah-Tikva, Israel

^2^ Department of Surgery, Sackler Faculty of Medicine, Tel Aviv University, Israel

^3^ Department of Imaging, Rabin Medical Center, Petah-Tikva, Israel.

^4^ Division of Imaging, Shaare Zedek Medical Center, Jerusalem, Israel

^5^ Department of Pathology, Rabin Medical Center, Petah-Tikva, Israel.

**Supplementary data**

**Table of contents:**

| Supplement | Content | page |
| --- | --- | --- |
| Supplementary Figure legend |  | 2 |
| Table S1 | Characteristics of all randomized participants at baseline | 3 |
| Table S2 | Cancer detection stratified by the biopsy approach taken first | 4 |
| Table S3 | Cancer detection in non-index MRI lesions and systemic cores | 5 |

Figure S1: Subgroup analyses of biopsy results within selected baseline categories.

csPCa – clinically significant prostate cancer; PI-RADS - Prostate Imaging Reporting and Data System; PSA – Protein Specific Antigen; TP – transperineal; TR – transrectal

Table S1 – Characteristics of all randomized participants at baseline

|  | All  (N=82) |
| --- | --- |
| Age years, median (IQR) | 68 (63-71.8) |
| PSA ng/ml, median (IQR) | 8.6 (6.2-12.2) |
| Prostate volume cm^3^,  median (IQR) | 54 (40-82) |
| PSA density, median (IQR) | 0.1 (0.09-0.26) |
| Biopsy Naïve, No. (%) | 10 (12%) |
| Previous negative biopsies, No. (%): |  |
| 1 | 23 (28%) |
| 2 | 17 (21%) |
| 3 and up | 7 (9%) |
| Active surveillance, No. (%)^a^ | 25 (30%) |
| Family history of PCa, No. (%) | 13 (16%) |
| PI-RADS No. (%) |  |
| 3 | 37 (45%) |
| 4 | 35(43%) |
| 5 | 10 (12%) |
| Location along axial view, No. (%) |  |
| Apex | 43 (52%) |
| Base | 10 (12%) |
| Midgland | 29 (35%) |
| Location along cornal view, No. (%) |  |
| Peripheral | 50 (61%) |
| Transition zone | 18 (22%) |
| Anterior | 14 (17%) |
| Ellipsoid index lesion volume ^b^,  median [IQR] | 0.38 (0.17-1.06) |

^a^ All participants under active surveillance protocol has previous biopsy with GG1.

^b^ Ellipsoid index lesion volume = 4Π/3 * Height * Length * Width

PCa – Prostate Cancer; PI-RADS - Prostate Imaging Reporting and Data System; PSA – Protein Specific Antigen

Table S2 – Cancer detection stratified by the biopsy approach taken first

|  |  | Arm1:  TP-fusion first  TR-fusion second  (N=39) | Arm2:  TR-fusion first  TP-fusion second  (N=38) | p-value |
| --- | --- | --- | --- | --- |
| Total biopsy results  n (%) | csPCa | 16 (41%) | 16 (42%) | *0.92 ^a^* |
|  | All PCa | 25 (64%) | 22 (58%) | *0.58 ^a^* |
|  | No Cancer | 14 (36%) | 16 (42%) | *0.58 ^a^* |
|  | GG1 | 12 (31%) | 11 (29%) | *0.22^b^* |
|  | GG2 | 5 (13%) | 9 (24%) |  |
|  | GG3 | 4 (10%) | 0 (0%) |  |
|  | GG4 | 2 (5%) | 1 (3%) |  |
|  | GG5 | 2 (5%) | 1 (3%) |  |
| TP-fusion index lesion biopsy results  n (%) | csPCa | 16 (41%) | 16 (42%) | *0.92 ^a^* |
|  | All PCa | 23 (59%) | 21 (55%) | *0.74 ^a^* |
|  | No Cancer | 16 (41%) | 17 (45%) | *0.74 ^a^* |
|  | GG1 | 10 (26%) | 12 (32%) | *0.29 ^b^* |
|  | GG2 | 5 (13%) | 7 (18%) |  |
|  | GG3 | 4 (10%) | 0 (0%) |  |
|  | GG4 | 2 (5%) | 1 (3%) |  |
|  | GG5 | 2 (5%) | 1 (3%) |  |
| TR-fusion index lesion biopsy results  n (%) | csPCa | 12 (31%) | 8 (21%) | *0.33 ^a^* |
|  | All PCa | 16 (41%) | 16 (42%) | *0.92 ^a^* |
|  | No Cancer | 23 (59%) | 22 (58%) | *0.92 ^a^* |
|  | GG1 | 6 (15%) | 10 (26%) | *0.11 ^b^* |
|  | GG2 | 7 (18%) | 4 (11%) |  |
|  | GG3 | 0 (0%) | 2 (16%) |  |
|  | GG4 | 2 (5%) | 0 (0%) |  |
|  | GG5 | 1 (3%) | 0 (0%) |  |

In this table we show that the order of approaches (i.e. which approach was performed first) had no impact on cancer detection rates.

TP-fusion identified total of 32 csPCa cases (42%). 16 cases were detected by the TP-fusion, when it was done first (41%) and 16 cases when the TR-approach was done first (42%) – showing that the detection rate of the TP was not influenced by order of approach.

TR-fusion csPCa detection rate (20 cases; 26%) was not influenced by order of approaches: 12 cases (31%) when TP was first, and 8 cases (21%) when TR was first.

^a^ p-value was calculated by Z-Score for 2 Population Proportions

*^b^* p-value was calculated by Fisher test

csPCa – clinically significant prostate cancer; GG – grade group; TP – transperineal; TR - transrectal

Table S3 – Cancer detection in non-index MRI lesions and systemic cores

|  | Non-index lesions  (N=61) | Systemic  (N=77) |
| --- | --- | --- |
| Biopsy outcome, n (%) |  |  |
| No Cancer | 45 (73.8%) | 69 (89.6%) |
| GG1 | 12 (19.7%) | 6 (7.8%) |
| GG2 | 3 (4.9%) | 2 (2.6%) |
| GG3 | 0 (0%) | 0 (0%) |
| GG4 | 0 (0%) | 0 (0%) |
| GG5 | 1 (1.6%) | 0 (0%) |
| csPCa detection, n (%) | 7 (11.5%) | 2 (2.6%) |
| Total cancer detection,  n (%) | 16 (26.2%) | 8 (10.4%) |
| GG2 and up, n (%) | 4 (6.6%) | 2 (2.6%) |
| GG3 and up, n (%) | 1 (1.6%) | 0 (0%) |

csPCa – clinically significant prostate cancer; GG – grade group;

TP – transperineal; TR – transrectal
