# Supplementary figures and images for: A noninferiority within-person study comparing the accuracy of transperineal to transrectal MRI–US fusion biopsy for prostate-cancer detection
Source: Prostate Cancer Prostatic Dis. 2020 Jan 17;23(3):449–56. doi: 10.1038/s41391-020-0205-7 (PMC7423592; doi:10.1038/s41391-020-0205-7)

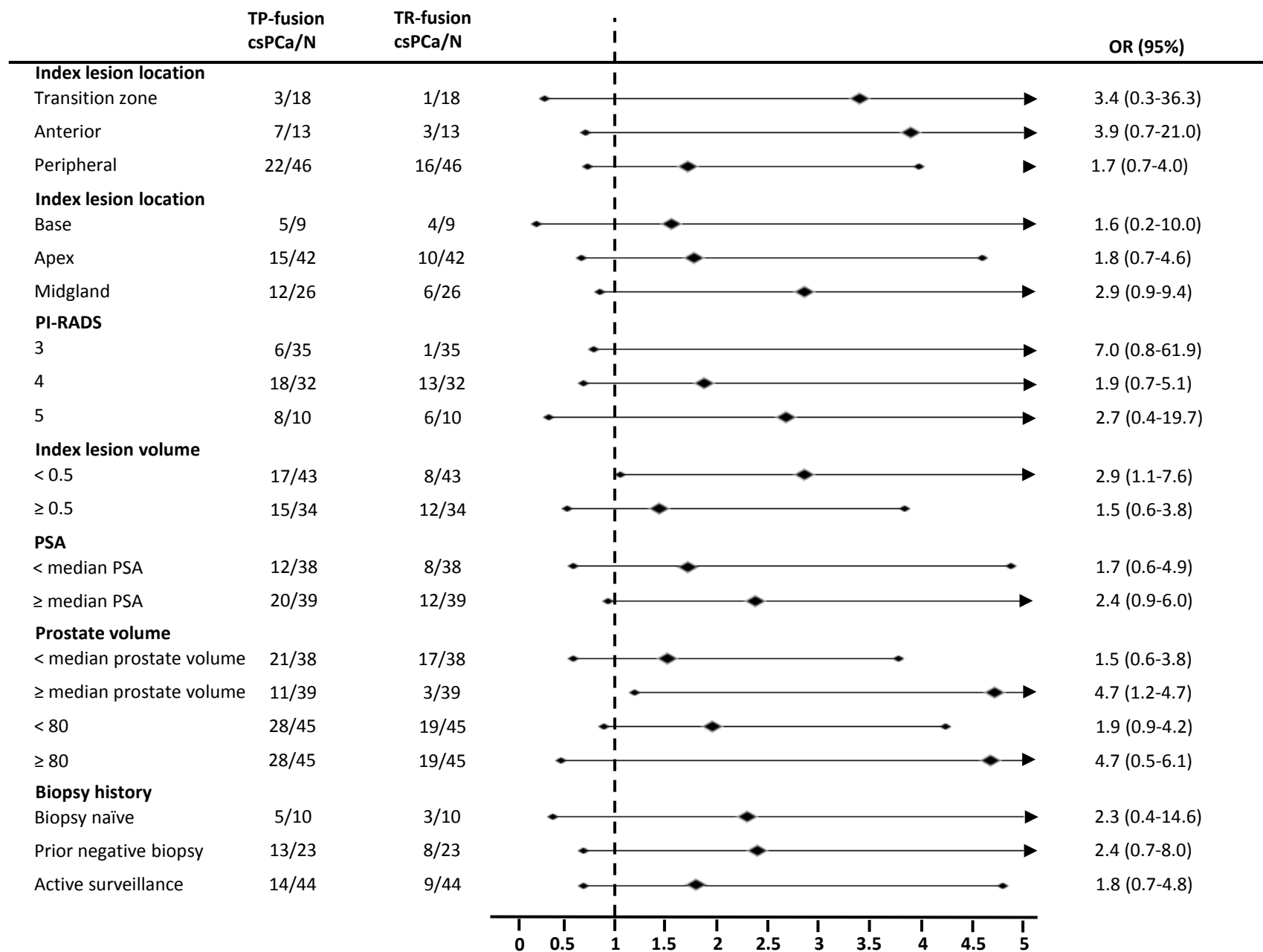

Supplement: Supplementary file 2 — Supplementary Figure S1 [file 41391_2020_205_MOESM2_ESM.pdf]
